# FabNet: A Features Agglomeration-Based Convolutional Neural Network for Multiscale Breast Cancer Histopathology Images Classification

**DOI:** 10.3390/cancers15041013

**Published:** 2023-02-05

**Authors:** Muhammad Sadiq Amin, Hyunsik Ahn

**Affiliations:** Department of Robot System Engineering, Tongmyong University, Busan 48520, Republic of Korea

**Keywords:** artificial intelligence, deep learning, pattern recognition, computer-assisted diagnosis, convolutional neural networks, breast cancer, colon cancer, histopathological images

## Abstract

**Simple Summary:**

Histology sample images are usually diagnosed definitively based on the radiologist’s extensive knowledge, yet, owing to the highly gritty visual appearance of such images, specialists sometimes differ on their evaluations. Automating the image diagnostic process and decreasing the analysis time may be achieved via the use of advanced deep learning algorithms. Diagnostic objectivity may be improved with the use of more effective and accurate automated technologies by lessening the differences between the humans. In this research, we propose a CNN model architecture for cancer image classification by accumulating layers closer together to further merge the semantic and spatial features. Regarding precision, our suggested cutting-edge model improves upon the current state-of-the-art approaches.

**Abstract:**

The definitive diagnosis of histology specimen images is largely based on the radiologist’s comprehensive experience; however, due to the fine to the coarse visual appearance of such images, experts often disagree with their assessments. Sophisticated deep learning approaches can help to automate the diagnosis process of the images and reduce the analysis duration. More efficient and accurate automated systems can also increase the diagnostic impartiality by reducing the difference between the operators. We propose a FabNet model that can learn the fine-to-coarse structural and textural features of multi-scale histopathological images by using accretive network architecture that agglomerate hierarchical feature maps to acquire significant classification accuracy. We expand on a contemporary design by incorporating deep and close integration to finely combine features across layers. Our deep layer accretive model structure combines the feature hierarchy in an iterative and hierarchically manner that infers higher accuracy and fewer parameters. The FabNet can identify malignant tumors from images and patches from histopathology images. We assessed the efficiency of our suggested model standard cancer datasets, which included breast cancer as well as colon cancer histopathology images. Our proposed avant garde model significantly outperforms existing state-of-the-art models in respect of the accuracy, F1 score, precision, and sensitivity, with fewer parameters.

## 1. Introduction

Breast cancer is the most prevalent types of cancer in women, affecting 2.1 million women annually, and it is responsible for the bulk of cancer-related deaths globally [1]. It has been estimated that the prevalence rates of breast cancer range from 19.3 per 100,000 African women to 89.7 per 100,000 European women [2]. Breast cancer is a fatal condition that can occur in nearly any bodily region or tissue when irregular cells abnormally spread, infiltrate, or move into adjacent tissues. The number of reported cases has increased in recent years, and it is projected to reach 27 million by 2030 [3,4,5,6,7]. Considering the high cancer mortality rate, colonoscopy and computer tomography are recommended for regular tests [8]. A biopsy examination is used to diagnose abnormalities in the breast and colon if suspicious cells are found. Hematoxylin and eosin (H&E) are often used to stain the isolated sample. When Hematoxylin interacts with Deoxyribonucleic Acid (DNA), it dyes the nuclei purple or blue, while Eosin stains other structures pink when it reacts with proteins [9].

The diagnosis of all cancer types, including breast and colon cancers, is based on histopathological images, which are considered to be essential. Histopathological examination, contrastingly, is a long-winded clinical practice, with the key impediment to successful image processing being a difference in the visibility in th H&E-colored regions. Various considerations, such as laboratory technique anomalies, discrepancies in sample positioning, operator-related heterogeneity, device diversity, and the usage of different fluorophores for staining, may all influence the diagnosis [10]. For even seasoned oncologists, recognizing and evaluating these discrepancies during a diagnosis could be challenging. As a result, there is a significant necessity for intelligent automated diagnostic systems to provide oncologists with reliable evaluations and improve the diagnostic performance.

Deep-learning-based approaches are currently the course of the research, and they have a profound impact on clinical trials and even the evolution and progress of targeted treatment methods. With the advancement in digital imaging technology, the automated diagnosis and detection of cancer types in whole slides images have received a great deal of interest. Several methods for analyzing histological images have been adopted, ranging from conventional to machine-learning-based ones [11]. Deep learning (DL) approaches have increasingly outperformed traditional machine learning (ML) algorithms in terms of end-to-end processing automation [12,13]. Deep learning-based techniques such as convolutional neural networks (CNN) have been successfully used in medical imaging to detect diabetic retinopathy [14], diagnose bone osteoarthritis [15], and for other purposes [16]. CNN-based histological image analysis methods have previously been shown to be effective for breast cancer diagnosis [17] and micro-level pathological image analysis [18,19].

The advent of the use convolutional neural networks as the basis of several visual tasks for different applications has made architecture searching a key driver in sustaining advancement with the right task extensions and data [20,21,22]. Because of the growing size and sophistication of networks, more effort is being put into developing the architecture motifs of nodes and nodes connectivity strategies that can be integrated systematically. This has resulted in wider and deeper networks; however, there is a need for more closely linked networks. To overcome these obstacles, various blocks or units have been integrated to match and change the network sizes, such as bottlenecks for reducing the dimensions [23,24] or residual, concatenated connections for features propagation [25,26].

In this paper, we suggest a CNN model design by accumulating layers that are even more close together to further fuse the semantic and spatial details for cancer image classification. Our accretive architecture incorporates more depth and sharing by expanding the existing approaches’ “shallow” skip connections [27] and focuses on merging the features from all of the layers and channels. Our contributions to this research are as follows:

1. We proposed a FabNet model that can learn the fine-to-coarse structural and textural features of multi-scale histopathological images by accretive network architecture that agglomerate hierarchical feature maps to acquire significant classification accuracy.
2. To preserve and integrate the features, our model links convolutional blocks in a closely coupled tree-based architecture. This method employs every layer of the network from the shallowest to the deepest layers to learn about the rich patterns that occupy a large portion of the feature pile.
3. We assessed the FabNet model using two publicly available standard datasets that are related to breast cancer and colorectal cancer and noticed that it outperforms the current state-of-the-art models in terms of accuracy, F1 score, sensitivity, and precision when we evaluated our model at different magnification scales of both binary and multi classification.

The rest of this article is structured as follows: Section 2 addresses the related work. Section 3 defines the design of the proposed FabNet model. We define the experimental setup, datasets, training, and implementation descriptions, and provide a detailed analysis of the performance in Section 4. The discussion, conclusions, and possible future research directions are all contained in Section 5.

## 2. Related Works

There has been extensive work that has been conducted in the literature to establish strategies for classifying and recognizing breast and colon cancers from histopathology images. The majority of the current approaches utilize computer-aided diagnosis (CAD) techniques to identify breast-cancer-related tumors that include benign and malignant ones. Before the deep learning breakthrough, the data were examined using conventional machine learning techniques based on supervised learning methods [28] to obtain the data features.

### 2.1. Conventional Learning Methods

The bulk of the research in this area has concentrated on a small data sample taken mostly from proprietary datasets. In 2013, several algorithms were used to classify the nuclei from a dataset containing five hundred images from fifty patients, including Gaussian mixture models and fuzzy C-means clustering techniques. This study reported 96% accuracy for two category classifications [29], suggesting that such machine learning-based approaches allowed adequately comprehensive and precise research and were considered to be useful for supporting breast cancer diagnostics. Spanhol et al. [30] published yet another study in which they achieved 85.1 % accuracy on a breast cancer dataset. They applied support vector machines for a patient-level analysis. Using a database of ninety-two specimens, George et al. [31] proposed a breast cancer classification method by applying neural nets with a support vector machine, which achieved 94 percent accuracy. Zhang et al. [32] suggested a cascading approach with a refusal alternative. This procedure was evaluated on a dataset with 361 specimens [33]. This study [34] suggested the application of different classifiers such as support vector machines and the k-nearest neighbor for breast cancer histology image classification. They achieved 87 % accuracy by utilizing assembling voting using the mentioned techniques. In this study [35], adaptive sparse support vector machine-based techniques were applied on a dataset at a 40× magnification level. They reported 94.97% accuracy. There have been a couple of other studies on histopathological representations for carcinoma classification; these studies specifically explain the dichotomies and shortcomings of various publicly accessible benchmark data [36,37].

### 2.2. Deep Learning Approaches

Deep learning has ushered in a new era in the domain of general object classification and detection. The classification of cancer histopathological images (i.e., breast and colon) has been a significant field of study due to advances in medical computer vision and deep learning. Because of the elevated histopathological image resolutions, the conventional machine learning algorithms and deep neural network models used to explicitly view the WSI have resulted in very complex network designs that are a challenge to training [38]. The number of samples used in the classification cancer histopathology images is limited, and the image size is large, making the training of CNN models challenging. Furthermore, image compression of the entire oncology image array to the CNN’s input size would result in a loss of the richness of the detailed feature data. As a result, some researchers suggested the classification of images based on patches to alleviate the challenge. In this study [39], the author used a technique to achieve the arbitrary extraction of patches based on a window slithering approach to extract image patches from the BreakHis dataset. AlexNet [40] was trained on the extracted patches, and then, integrated the outcomes to classify into relevant categories. Another study by Arajo et al. [3] suggested a convolutional neural network for automatic feature extraction from a dataset that contained 512 × 512 size patches. The images were grouped into four classes during training, which were used for multi-classification, as well as two classes, which were used for binary classification. 

Because of the image patches extraction process, CNN became capable of training whole slide images with reasonable details. This study [41] suggested a convolutional neural network with a two-level model for high-resolution WSIs classification. The first model is based on a minimal anomaly model that can distinguish between patterns automatically during training on image patches, and a second model that classifies the results by an SVM classifier. In another study, Alom et al. [2] suggested the merging of three models to classify breast cancer histology images. A CNN-based methodology achieved 77.8 percent accuracy for multi-classification, while it found an 83.3 percent accuracy for binary classification on the breast histology 2015 dataset [3].

Han et al. [42] recently suggested a class structure-based deep convolutional neural network that achieved 93.2 percent accuracy on the BreakHis dataset. Table 1 elaborates on the details of recent advancements in the cancer research domain.

Even though the preceding studies demonstrate that patch-based image classification approaches are commonly used in different breast cancer histopathology datasets, histopathology images contain a large number of fine details that need to be extracted with utmost accuracy and precision. We present FabNet, a CNN model that ensembles every fine-to-coarse detail for more accurate learning. This method employs every layer of network from the shallowest to the deepest layers to learn about the rich patterns that occupy a large portion of the feature pile.

## 3. FabNet: Features Agglomeration Approach

**Figure 1:**
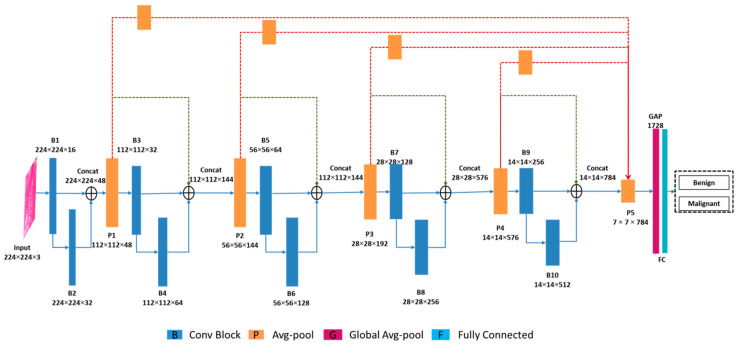
FabNet model: a detailed architectural overview.

**Figure 2:**
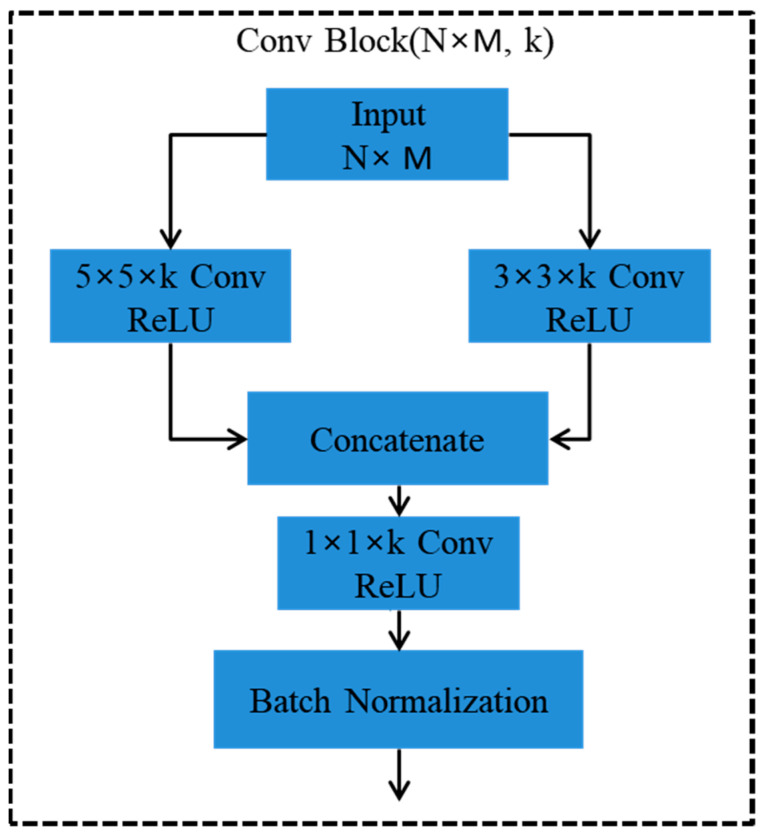
Internal architecture of conv blocks (i.e., B1). The input passes through two 5 × 5 and 3 × 3 convolutional layers; the output is concatenated in the proceeding step.

We define agglomeration as the combination or merging of network layers in a closely coupled manner. In the proposed model FabNet, as shown in Figure 1, we are particularly focused on the productive accumulation of depth, dimensions, and resolutions. We define an agglomeration sequence as deep if it is holistic, discrete, and the initial agglomerated layer moves features through several agglomerations. Since our network has multiple layers and connections, we designed modular architecture that tends to reduce the complexity by grouping and replication. The proposed network layers are subdivided into blocks, for example, B1, which are further subdivided into stages based on the feature resolution. This design is focused on agglomerating the blocks to preserve and combine the feature channels. In Figure 2, a conv block (i.e., B1) is shown, which comprises two convolutional layers with 5 × 5 and 3 × 3 filter window sizes. Both of the convolutional layer activation maps are concatenated, and then transferred to another convolutional layer with 1 × 1 filter size window to reduce the optimal channels. Agglomeration starts on the smallest, shallowest scale and gradually merges on the deeper, wider scales in a repetitive manner. In this manner, the shallow features are redefined as they progress over to deeper blocks of layers.

For a sequence of blocks $\left\{B1,B2,B3\ldots ..Bn\right\}$, we formulated the function ℜ for such a repetition below.

(1) $$ℜ\left(B1,B2,B3\ldots ..Bn\right)=ℜ\left(\Sigma B1,B2,B3\ldots ..Bn\right)$$

In Equation (1), n is the number of blocks. To increase the depth of the network and the performance, we merge or fuse blocks in a tree-like closely coupled structure. We pass an agglomerated node’s feature map back to the baseline as the input feature map to the next sub-module, instead of forwarding intermediate agglomerations further up the tree. This spreads the agglomerations of all of the previous modules, rather than the preceding module only, to help the best preservation of features. We combine the parent and left child nodes of the same depth in the performance.

Our model consists of conv blocks, which are the basic building block of each node. The input of a conv block in the case of B1 accepts an input of 224 × 224 × 3. This input is passed to two different convolutional layers simultaneously for convolutional operations to be performed. Both of the convolutional layers apply 16 kernels with filter window sizes of 3 × 3 and 5 × 5 each with nonlinearity (ReLU), which aims to alleviate the issue of vanishing gradients, as well as improve the network’s training speed. To generate an optimal feature map, the feature maps of these two convolutional layers are combined, and thereafter, transferred to a 1 × 1 convolutional layer. In each convolutional layer that is discussed above, we use zero padding, which preserves the original image size, while also providing valuable knowledge about feature learning, which aids in the extraction of low-level features for the subsequent layers. Following that, we apply batch normalization, which balances the inferences of the preceding activation layer by subtracting the batch mean and dividing the batch division, thereby increasing the network stability.

The output of conv block B1 is fed into B2, which has a similar internal architecture to that of B1, as depicted in Figure 2, except for the number of kernels. Conv B2 contains 32 convolution filters. The feature maps of both of the conv blocks are then concatenated, which results in an enhanced collective feature map. We apply an average pooling operation with an average pooling layer with 2 × 2 patches of the feature map with a stride of two. This layer down-samples the estimation complexities and parameters from the evaluated image by dividing it into rectangular pooling window areas, which is proceeded by a mean value estimation for every region. The inference of the average pooled image propagates to the next block as an input to conv block B3, which is fed into the final stage C5. As it was mentioned earlier, B3 contains the same internal architecture as those of conv blocks B1 and B2, but the number of convolution filters is 32. The output feature map of B3 is fed into conv block B4 as an input. The internal convolutional layers of conv block B4 apply 64 convolution filters to learn the features. The feature maps of B3 and B4 are fused to generate an extended feature map, which is proceeding by average pooling for down-sampling. The average pooled value feeds into the next conv block B5. Conv block B6 is fed to B5 as an input. B6 utilizes 128 convolution filters. 

The feature maps of conv block B5 are conv block B6 which is concatenated to fuse the feature, which results in an enhanced feature map with detailed data information. This step is preceded by an average pooling operation to obtain half of the image size. The result of the pooled value is fed into conv block B5. The network repeats the same operation until it reaches conv block B10. The only difference is between the blocks is the number of convolution filters, which is 256 for B8 and 512 for B10. Until it reaches B10, the feature maps of the entire network resulted in optimized propagation from the shallower to the deeper layers and blocks, which makes the proposed network compact and closely bind the entire network. The best features of every block and stage are collected and fused at stage C5 by the extensions from C1 to C5 and by bridging the adjacent blocks The C5 is subjected to the global average pooling function, which significantly reduces the number of data, and thus, the classification layers by measuring the average results of every feature map in the preceding layer. The output layer, which is the last dense layer, includes neurons for each class that have been normalized with the Softmax function; the amount of them varies based on the classification category. We used binary and multi-class classifications in this study.

## 4. Methodology

**Figure 3:**
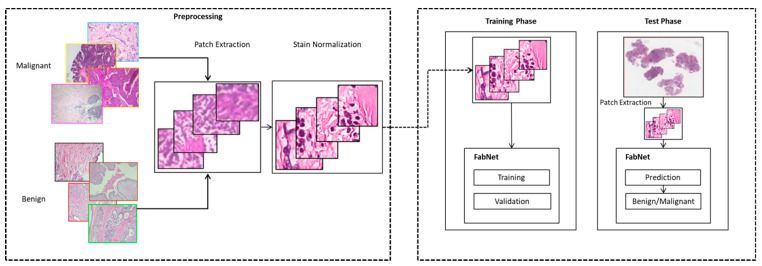
An overview of the proposed methodology to classify the histopathological image.

As seen in Figure 3, the proposed method consists of three main steps. Firstly, we obtain training samples by applying the extraction of patches technique to the dataset. Secondly, stain normalization preprocessing of the dataset is performed to resolve the stain variation in the images. For stain normalization, several methods have been suggested in these studies [49,50,51]. DL-based approaches for classifying cancer histopathology images employs a training set to detect a wide range of enhancements to distinguish variations within, as well as across, the categories. A wide range of color inconsistencies in the histopathological images may occur due to the color response of the automated scanners, stain supplier materials and processing units or due to various staining procedures in different laboratories. Therefore, stain normalization is a basic step during histopathological image preprocessing. The key benefit of using image patches for each type of training is that it preserves the local characteristic information from the histopathology images, helping the model to learn the local characteristics features. Thirdly, we train our proposed model with these extracted images to classify and differentiate between the benign and malignant tumors. Furthermore, we outline the datasets, image preprocessing, model training, and implementation details below.

### 4.1. Dataset

To evaluate our proposed model, we used the two main, public cancer histology image datasets. Such datasets were considered with three motives: firstly, the diversity of cancer types represented in the histology slides, such as breast cancer and colorectal cancer; secondly, their amount; thirdly, the existence of multiple magnification factors that helped us to carry different tests with the restricted equipment, while modifying different parameters.

#### 4.1.1. BreaHis

**Figure 4:**
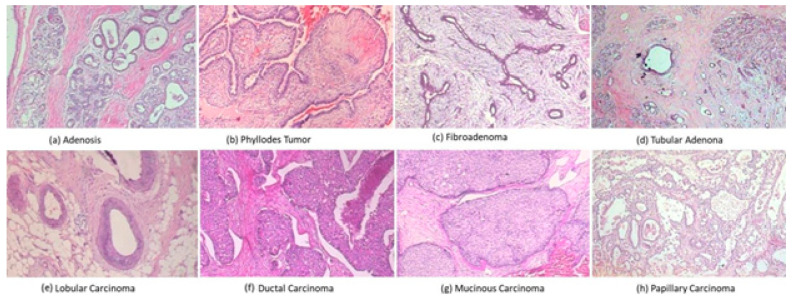
From the BreakHis dataset, the first row depicts benign 4 subclasses, while the second row shows malignant 4 subclasses. These images have a magnification factor of 200×.

In this study, we assessed our model with BreakHis, a publicly available breast-cancer-related histologic dataset [30]. Samples were created using breast tissue biopsy slides that were colored with H&E staining. There are reportedly 7909 histopathological biopsy images of 700 × 460 pixels in the BreakHis dataset from eighty-two individuals. The dataset consists of two main categories: one of them is benign, and the other one is malignant, which are further subdivided into 4 subclasses as per each category. Table 2 shows the statistical specifics of this dataset, and Figure 4, shows a few illustrations of the histological images. For our tests, we randomly divided the entire dataset in into training/testing subgroups at a 70:30 ratio. To assess our model’s efficiency in clinical settings, we kept a patient-based distinction between the training and test data. For stain normalization, we adopted the technique suggested in [50]: an innovative composition-preserving color normalization (SPCN) scheme is used in this process.

**Figure 5:**
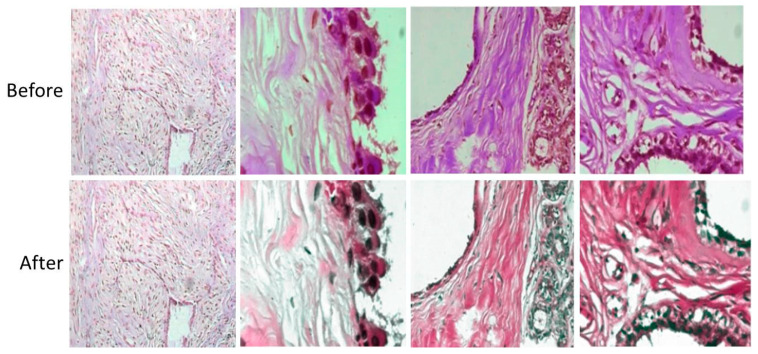
Stain normalized images of 4 different subcategories at a magnification factor of 400×.

The illustration of stain normalized images is shown in Figure 5.

#### 4.1.2. NCT-CRC-HE-100K

**Figure 6:**
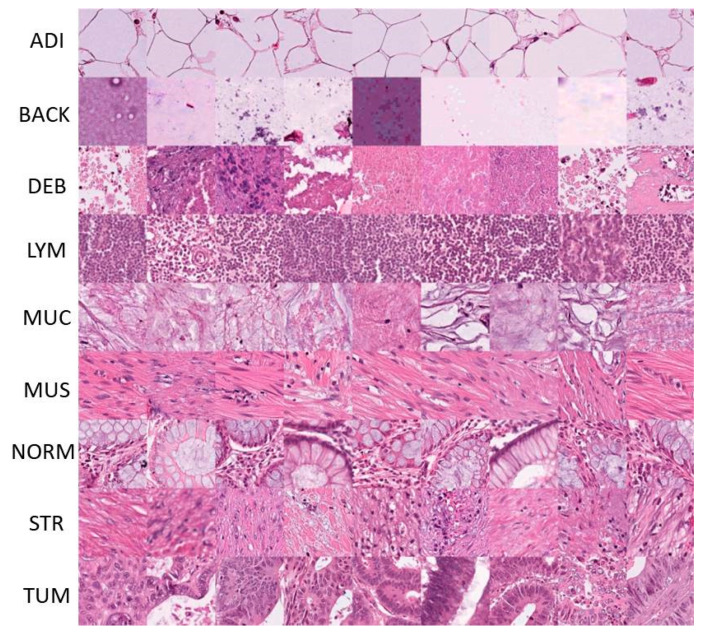
An illustrative image from nine classes of human colorectal cancer datasets.

This dataset includes publicly available 100 K images of human colorectal cancer (CRC), as well as normal tissues [52]. To stain normalize this dataset, in which the image size was 224 × 224 pixels, the Macenko approach [53] was used. We used this color normalization technique because the initial images had subtle variations between red and blue tones, resulting in a misleading classification. Figure 6 shows descriptive representations of the sample images. This dataset is divided into nine subclasses, which are adipose tissue (ADI), lymphocytes (LYM), background (BACK), mucus (MUC), smooth muscle (MUS), normal (NORM), debris (DEB), cancer-associated stroma (STR), and tumor (TUM) ones. To improve the variance in this training set, normal tissue samples were obtained primarily from clinical specimens, as well as from gastrectomy samples (such as upper gastrointestinal smooth muscle). The number of distributed training set images in each group was nearly equal, while the test samples contained 7180 images.

### 4.2. Image Representation and Patch Extraction

Table 2 shows that the BreakHis dataset has a data imbalance problem, which was calculated as 0.42 at the case image scale and 0.44 at the patient scale. The data disparity problem can cause a discriminating performance of computer-aided diagnosis (CAD) models against the majority class in classification problems. Equation (2) determines the patch amount obtained from the dataset image of the ith class.

(2) Ni=⌈∑i=1nxin|xi×β⌉

Equation (2) depicts a mathematical representation of N_i_ patches derived from the i(th) category, x_i_ is the i(th) category’s number, x_th_ is the i(th) category’s number, β is a constant value, and n represents the classes. The fixed parameter (β) was set to 32. After that, each class has nearly the same number of patches. The primary benefit of utilizing patches during training for every individual class is that it preserves the regional distinctive details in the histological image, which enables the model to learn the spatial information [54]. 

To obtain an image classification, first, we use a patch classifier to compare several distinct magnifications of patches, and afterward, we average the effects for the complete image patches. The extraction and learning of similar features, for instance, the entire tissue composition, nucleus state, and texture features are used to classify the images to the desired categories. We inferred that 224 × 224, as well as 700 × 460-pixel patches, would be sufficient to justify the proper cell formation of various tissues. We deduced that 700 × 460, as well as 224 × 224 px size for images, would be ample to explain the relevant composition of different tissues.

## 5. Experimental Results

### 5.1. Model Training

We assessed the proposed model’s efficiency in two areas: (1) sample classification based on binary and multi-class classification, and (2) sample classification based on patient- and image-level classification. We used the datasets discussed in the study. These datasets were subdivided into training validation sets. To find the optimal parameters for our model, we use a five-fold cross-validation scheme. We assess our model with assessment metrics such as accuracy, sensitivity, and precision, and F1 score in the performance assessment. On an NVIDIA GTX 1080Ti, we used the Keras framework to implement the method. The metrics of five successful completed trial experiments are reported. We compared our model’s efficiency to that of cutting-edge models such as DenseNet 121 [55], VGG16 [56], and ResNet 50 [57].

### 5.2. Implementation Details

FabNet model assimilates the fine-to-coarse structural and textural features of multi-scale histopathological images by accretive network architecture that agglomerate hierarchical feature maps to perform significant learning. Our model propagates the features from block to block, and overall, from stage to stage to ensemble the best feature map for learning. We tuned the following hyperparameters in our model, which are a number of convolutional blocks (the internal architecture is defined in Figure 2), epochs, learning rate, optimizer, size of batch, and batch normalization. The epochs were set to 20, 50, 70, and 100, respectively, while 0.01, 0.001, 0.0001, and 10^−4^ learning rates were evaluated. We used a batch size of 16, 32, and 64 due to hardware limitations. We tested the model with different optimizers such as Adadelta, Adamax, SGD, RMSprop, and Nadam, but Adam provided the optimal accuracy. The detailed optimized hypermeters are shown in Table 3.

The proposed BreakHis and NCT-CRC-HE-100K datasets intended to serve as a standard for breast and colon cancer CAD systems. Before discussing the results, we define the evaluation matrices, which were used to assess the proposed model. The experimental procedure for evaluating the proposed approach for the BreakHis dataset is similar to that which was used in the previous study [39]. The authors defined two types of accuracies, in which the first one reflects the performance accuracy achieved on the patient scale. 

If we suppose *N_p_* represents the images of the patient, while *N_c_* is the patient images that are accurately categorized and *N_t_* are the total patients, the score for an individual patient can be calculated as

(3) $$Patient\;Score=\frac{\;N_{p}}{N_{c}}\;$$

While the global patient accuracy can be calculated as,

(4) $$Patient\;Level\;Accuracy=\frac{\sum Patient\;Score}{N_{t}}$$

The second case for the evaluation of classification accuracy is image-level accuracy. If we let *N_tb_* be the test image samples for breast cancer and *N_cb_* be the images that are classified by CAD system accurately, according to labeled classes, the image level accuracy can be defined as follows,

(5) $$Image\;Level\;accurcy=\frac{N_{tb}}{N_{cb}}$$

The obtained accuracy at the image and patient levels for different magnification levels is shown in Table 4. Largely, a malignant case is considered to be positive during cancer diagnosis, whereas a benign case is considered to be negative. In clinical diagnosis, sensitivity (also known as recall) is more significant for medical professionals. Therefore, in this study, the proposed model is evaluated based on metrics defined below,

(6) $$Precision=\frac{True\;Possitive\;}{True\;Possitive+False\;possitive\;}$$

(7) $$Recall=\frac{True\;Possitive\;}{True\;Possitive+False\;Negative\;}$$

(8) $$F1\;\mathrm{score}=2\times \frac{Precision\times Recall}{Precision+Recall\;}$$

Table 4 depicts the performance of the proposed model, which outperformed DenseNet 121 and MSI-MFNET in terms of test accuracy at each magnification level using the BreakHis dataset. The model showed superior test accuracy at 40×, 200×, and 400× magnifications. At the 100× magnification level, the model slightly lags behind Dense121, which achieves 90.21% accuracy at the patient level, while it achieves 92.71 for the image-level classifications. 

The experiments are performed largely focused on binary and multiclass classification. The patch-wise binary and multi-classification outcomes are shown in Table 5. The results are shown using important metrics such as test accuracy and sensitivity (recall) using the 200× magnified image patches. The results are compared with those of two benchmark models, which are DenseNet121 and MSI-MFNet. The experimental results that are obtained by the proposed FabNet were better than the mentioned models were, with a larger margin in terms of test accuracy for binary classification as well as multi-class classification.

In Table 6, the detailed results that are obtained from the proposed model are presented. It is evident that the model exhibited better accuracy for binary classification, as well as multi-classification at contrasting magnifications, for instance, 40×, 100×, 200×, and 400×. The model showed better performance for binary classification, for instance, the accuracy at the 40× magnification scale the model achieved 99 percent accuracy. The model showed better performance for many classed as well.

Table 7 depicts the classification results of the proposed FabNet for the NCT-CRC-HE-100 K dataset. It is evident that the model exhibited an outstanding performance in terms of test accuracy and sensitivity compared to those of the benchmark models such as VGG16, DenseNet 121, and ResNet50.

In Table 8, detailed class-wise scores for important matrices such as precision, sensitivity, and recall are given to elaborate the efficiency using the NCT-CRC-HE-100K dataset.

**Figure 7:**
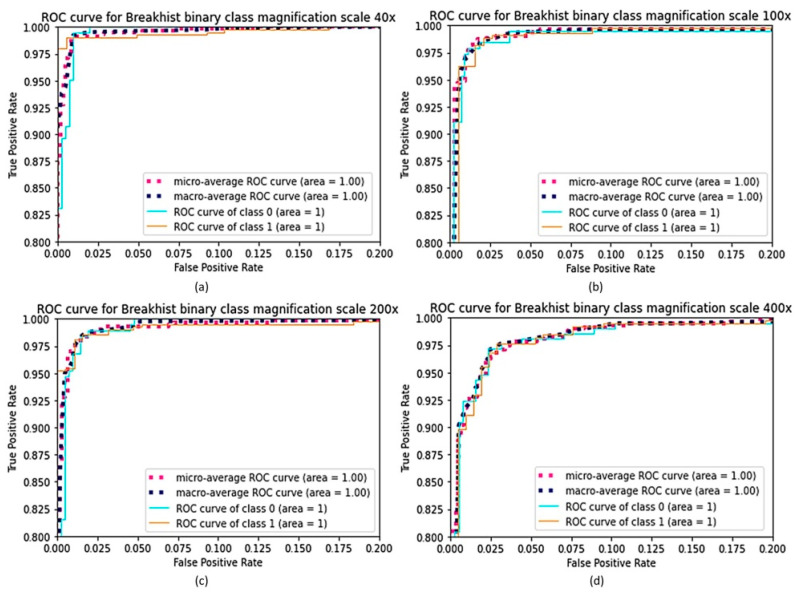
ROC curves of FabNet Model for binary classification, (**a**) 40× magnification, (**b**) BreakHis 100× magnification, (**c**) BreakHis 200× magnification, (**d**) BreakHis 400× magnification.

**Figure 8:**
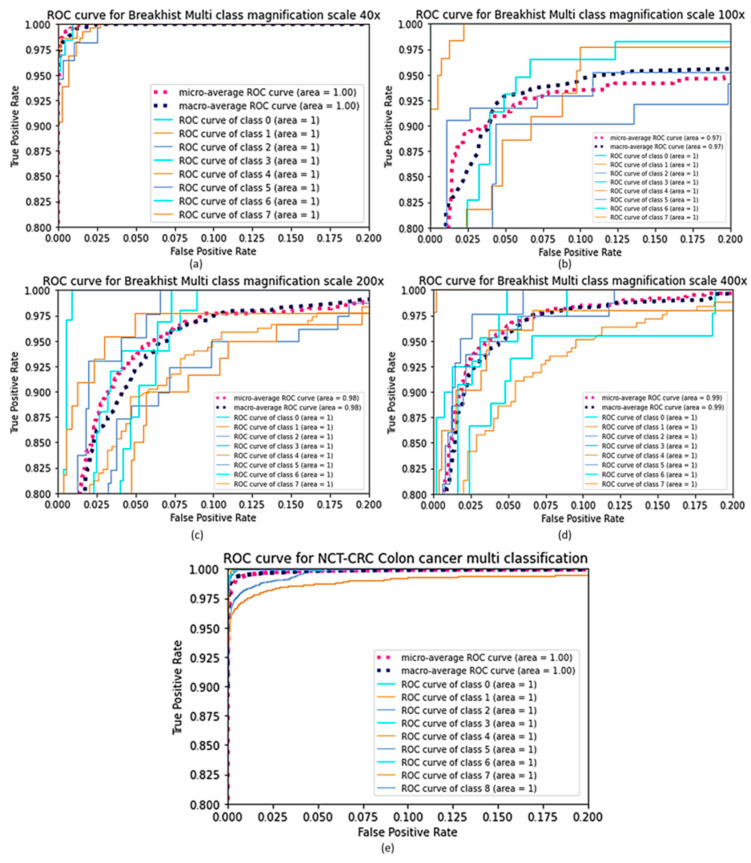
ROC curves of FabNet Model for multi classification, (**a**) 40× magnification, (**b**) BreakHis 100× magnification, (**c**) BreakHis 200× magnification, (**d**) BreakHis 400× magnification (**e**) ROC curves for NCT-CRC colon cancer dataset.

**Figure 9:**
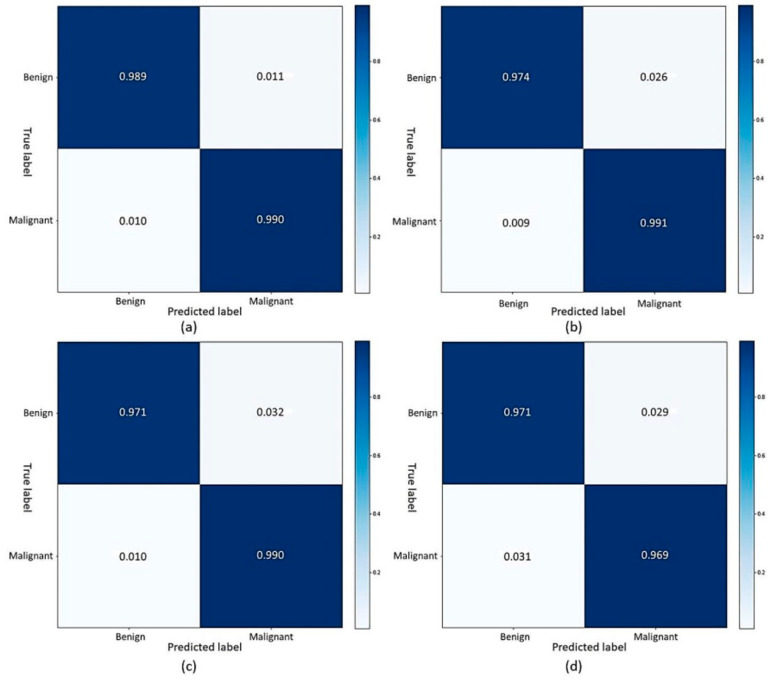
Confusion matrices of FabNet that for BreakHis, (**a**) confusion matrix of 40× magnification, (**b**) confusion matrix of 100× magnification, (**c**) confusion matrix of 200× magnification, (**d**) confusion matrix of 400× magnification.

The ROC curve is a graphical determination of the classification model’s results. It is determined by plotting the true positive rate (TPR) against the false positive rate (FPR) at various discriminatory thresholds, where TPR stands for sensitivity or recall, and FPR stands for false positive rate (1-specificity). The ROC curve for a classification algorithm would be a diagonal line from (0,0) to (1,1). Any curve above the diagonal line indicates a decent classification model that randomly outperforms, and any curve below the diagonal line indicates a model that randomly underperforms. The region under the ROC curve, which is often between 0 and 1, is referred to as the AUC. A high AUC means that the classification model is accurate according to the ROC curve concept. The ROC curve graph can be seen for the binary classification of the BreakHis dataset in Figure 6, where class 0 indicates a benign tumor, and class 1 represents a malignant tumor. Figure 7 and Figure 8 depict the ROC curve graph for the multi-classification performance using the BreakHis and NCT-CRC-HE-100K datasets. The confusion matrix for the binary classification of the BreakHis dataset at different magnification scales is shown in Figure 9. As can be seen in the cases of different magnification levels, 40×, 100×, and 200×, our model tends to produce better results for binary classification. Because of the diverse and significant areas in the images, the representation of the confusion matrix results shows that binary scenarios performed better than multi-classification scenarios did. The higher magnification of features give further structural information to the model, which helps it to acquire a decent depiction of patches with labels.

**Figure 10:**
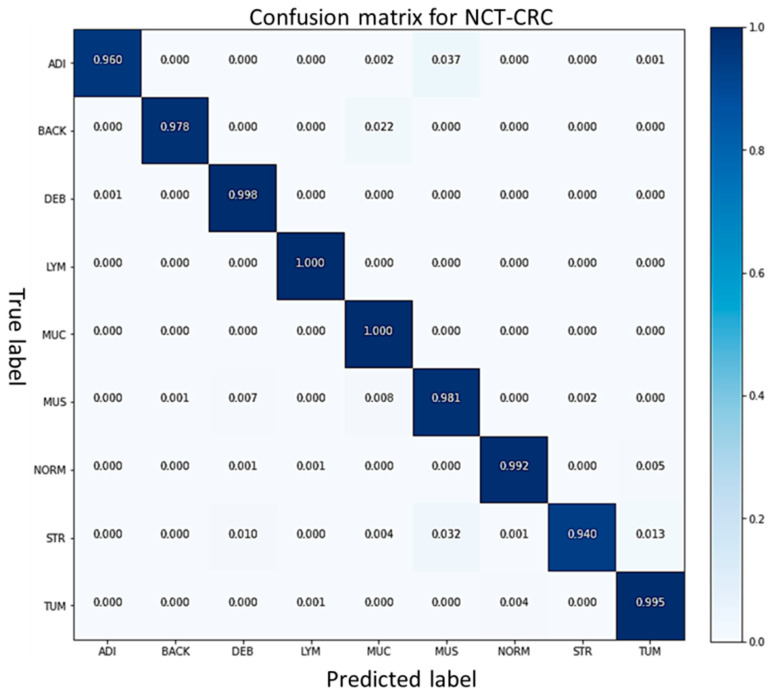
Confusion matrix of FabNet, which shows the best score in the NCT-CRC-HE-100K dataset testing set among 5-fold cross-validation.

The confusion matrix results for multi-classification in the case of NCT-CRC colon cancer are shown in Figure 10.

Table 9 and Table 10 shows the results of proposed model in comparison with benchmarks related to breast and colon histology models.

Table 9 shows the mean and standard deviation of our results by experimenting with satin and without stain normalization to better understand the use of the FabNet model in studying cancer histopathology images. 

The model outperformed some of the one in the most current research studies. For example, in [68], they obtained 97.58% and 97.45% accuracy rates with 7.6 million training parameters, whereas we reached a 99.03% accuracy with 3239 K training parameters. Despite having fewer training parameters, our model achieved a higher degree of accuracy. In another study [2], the authors proposed the Inception Recurrent Residual Convolutional Neural Network (IRRCNN) network, which obtained 97.95% accuracy for image classification and 97.65% accuracy for patient classification. Unlike IRRCNN, FabNet obtained a 99.01% patient-level accuracy and 99.03% picture-level accuracy using this dataset. The authors obtained 99.05% accuracy for binary classification and 98.59% accuracy for multiclassification using data augmentation. We obtained comparable outcomes without applying data augmentation. Data augmentation enables a learning model to overcome important training constraints such as overfitting, hence improving its accuracy and generalization capabilities. In the case of our model, we think that its ability for generalization is strengthened despite the absence of data augmentation. A similar accuracy was shown by Rui Man et al. [55] at the 40× magnification level, however our model achieved better results at the 200× and 400× magnification levels. The authors proposed the use of DenseNet121-ino, which has substantially more training parameters than FabNet does.

## 6. Conclusions

In this paper, we suggested the FabNet model that can learn the fine-to-coarse structural and textural features of multi scale histopathological images by accretive network architecture, which agglomerates hierarchical feature maps to acquire significant classification accuracy. We expanded upon the conventional convolutional neural network architecture by incorporating deeper integration to finely fuse information across layers. This layer expansion had a small impact on the model’s depth; however, it made the model more tightly linked with a compact form, ensuring that any piece of detail was transferred to the deeper layers for better learning. Despite having fewer parameters, this lightweight network architecture yielded better classification accuracy than the state-of-the-art models did. 

Our model yields improved classification probabilities at both the patch as well as the image levels. The efficiency and reliability of the FabNet were assessed using two public datasets that included breast and colon cancer data based on several experiments, for instance, multi- and binary classifications. The suggested FabNet improved upon the existing state-of-the-art models when they were evaluated using both of the public benchmark datasets. The experimental parameters were kept the same for the benchmark models, as well as for the proposed model to precisely conclude the performance. The proposed model achieved 99% accuracy and a 98.9% F1 score in the case of the binary classification of BreakHis at the 40× magnification scale. The model achieved 98.2% test accuracy and a 98.23% F1 score for NCT-CRC-HE-100K colon cancer dataset without employing any data augmentation technique. 

We believe that the model can reduce the cancer screening time for pathologists, as well as oncologists. In diverse circumstances, oncologists and researchers working in the field of cancer detection and diagnostics using histological images will benefit from the proposed model’s high sensitivity and accuracy. Although the closely coupled architecture tackled the imbalance in the dataset issue, which ultimately resulted in minor effects on the model’s performances, since the data imbalance is so prominent in the clinical histology, we intend to look at certain strategies for coping with this problem in the future. We will also look at which feature map combinations which are most significant for classification. The proposed model can be used to perform a variety of tasks related to histological image-based classification in clinical environments. 

## Figures and Tables

**Table 1:** A review of supervised learning models. The staining abbreviations stand for H&E (hematoxylin and eosin); PHH3 (Phosphohistone-H3).

Reference	Local/Global	Cancer Type	Staining	Method	Dataset
Ceresin et al. (2013) [43]	Local-level	Breast	Hematoxylin and eosin	CNN	ICPR2012 (50 images)
Wang et al. (2014) [44]	Local-level	Breast	Hematoxylin and eosin	Rippled integration of CNN	ICPR2012 (50 images)
Raza et al. (2016) [45]	Local-level	Colorectal	Hematoxylin and eosin	Cell detection Spatially constrained CNN + handcrafted features	Private CRC dataset (15 images)
Tellez et al. (2019) [46]	Local-level	Breast	Hematoxylin and eosin; PHH3	CNN	TNBC (36 images); TUPAC (814 images)
Ehteshami et al. (2017) [47]	Global-level	Breast	Hematoxylin and eosin	Stacked CNN incorporating contextual information	Private set (221 images)
Ehteshami et al. (2018) [48]	Global-level	Breast	Hematoxylin and eosin	Integration of DHACNN & LSTM	BreakHis (7909 images)

**Table 2:** BreakHis dataset categorization at patient level at four magnifications (40×, 100×, 200×, and 400×).

Category	Subtypes	Magnification				Sum	Individuals
		40×	100×	200×	400×		
Benign	Phyllodes Tumor (PHT)	149	150	140	130	569	7
	Fibroadenoma (FID)	253	260	264	237	1014	10
	Adenosis (ADE)	114	113	111	106	444	4
	Tubular Adenona (TUA)	109	121	108	115	453	3
Malignant	Papillary Carcinoma (PAC)	145	142	135	138	560	6
	Ductal Carcinoma (DUC)	864	903	896	788	3451	38
	Lobular Carcinoma (LOC)	156	170	163	137	626	5
	Mucinous Carcinoma (MUC)	205	222	196	169	792	9

**Table 3:** Optimized hyper-parameters for FabNet, Densenet121, DNet, VGG16, and ResNet50.

Dataset	Parameters	FabNet	DenseNet121	VGG16	ResNet50
BreakHis	Epochs	100	100	100	100
	Learning Rate	$10^{-3}$	$10^{-3}$	$10^{-3}$	$10^{-3}$
	Batch Size	16	16	16	16
	Number of layers	30	121	16	50
	Optimizer	Adam	Adam	Adam	Adam
	Number of parameters	3239 K	7138 K	14,765 K	23,788 K
NCT-CRC-HE-100K	Epochs	100	100	100	100
	Learning Rate	$10^{-3}$	$10^{-3}$	$10^{-3}$	$10^{-3}$
	Batch Size	64	64	64	64
	Number of layers	30	121	16	50
	Optimizer	Adam	Adam	Adam	Adam
	Number of parameters	3239 K	7138 K	14,765 K	23,788 K

**Table 4:** Performance comparisons in terms of accuracy for BreakHis dataset.

Accuracy (%)	Method	Magnification Level			
		40×	100×	200×	400×
Patient Level	DenseNet 121 [55]	92.02	90.21	81.94	80.09
	MSI-MFNet [58]	93.04	88.34	92.12	89.19
	Proposed FabNet	99.01	89.26	98.38	96.96
Image Level	DenseNet 121 [55]	94.26	92.71	83.90	82.75
	MSI-MFNet [58]	94.12	89.25	92.45	90.27
	Proposed FabNet	99.03	89.68	98.51	97.10

**Table 5:** Patch wise classification results of FabNet for BreakHis dataset on magnification level 200× in terms of accuracy and sensitivity metrics.

Class	Model	Accuracy	Sensitivity							
			Benign				Malignant			
Binary	DenseNet [55]	0.92	0.75				0.97			
	MSIMFNet [58]	0.92	0.76				0.98			
	FabNet	0.99	0.989				0.990			
			ADE	FIB	PHT	TAD	DUC	LOC	MUC	PAC
Multi	DenseNet121 [55]	0.84	0.60	0.84	0.72	0.84	0.86	0.85	0.97	0.91
	MSIMFNet [58]	0.88	0.60	0.87	0.79	0.89	0.96	0.75	0.98	0.92
	FabNet	0.97	1.00	0.88	1.00	1.00	0.804	0.89	0.784	0.865

**Table 6:** Detailed classification results of FabNet on BreakHis dataset based at different magnification levels.

Class	Magnification Level	Accuracy (%)	Precision (%)	Recall (%)	F1 Score (%)
Binary	40×	99.00	98.991	98.986	98.989
	100×	89.26	89.128	89.262	89.195
	200×	99.00	98.352	98.355	98.354
	400×	97.96	97.541	97.521	97.551
Multi	40×	91.26	90.635	89.126	88.289
	100×	97.00	96.531	96.427	95.912
	200×	97.05	85.972	85.526	85.748
	400×	97.20	89.947	89.851	88.899

**Table 7:** Detailed classification results by FabNet on NCT-CRC-HE-100K dataset concerning benchmark models in terms of accuracy and sensitivity.

Model	Accuracy (%)	Sensitivity								
		ADI	BACK	DEB	LYM	MUC	MUS	NORM	STR	TUM
VGG16 [56]	96.0	0.95	0.93	0.94	0.88	0.96	0.89	0.98	0.91	0.90
ResNet50 [56]	95.9	0.94	0.90	1.00	0.89	0.92	0.88	0.89	0.95	0.98
Dense Net 121 [55]	96.1	0.96	0.70	0.98	0.97	0.92	0.91	0.96	0.93	0.94
FabNet	98.2	0.96	0.98	1.00	1.00	1.00	0.98	0.99	0.94	0.99

**Table 8:** Class-wise results representation of FabNet in terms of precision, F1 score, and recall using the NCT-CRC-HE-100K dataset.

Class	Precision	F1 Score	Recall
Adipose Tissue	1.00	0.98	0.96
Background	1.00	0.99	0.98
Colorectal Cancer	0.98	0.99	1.00
Debris	1.00	1.00	1.00
Lymphocytes	0.95	0.97	1.00
Mucus	0.94	0.96	0.98
NC Tumor	0.99	0.99	0.99
Colon Mucosa	1.00	0.97	0.94
Cancer Stroma	0.99	0.99	0.99

**Table 9:** A comparison of FabNet performance with existing studies on BreakHis histology dataset.

Dataset	Author	Year	Preprocessing	Model	Accuracy (%) Magnification Level			
					40×	100×	200×	400×
Break his Dataset	Spanhol et al. [30]	2016	None	PFTAS QDA	83 ± 4.1	82.1 ± 4.9	85.1 ± 3.1	82 ± 3.8
	Spanhol et al. [39]	2016	Image Resize	Pre-Trained AlexNet	88 ± 5.6	84.5 ± 2.4	85.3 ± 3.8	81 ± 4.9
	Spanhol et al. [59]	2017	None	DeCAF Model	84 ± 6.9	83.9 ± 5.9	86.3 ± 3.5	82 ± 2.4
	Kumar et al. [60]	2018	Image Resize	Newly Designed CNN	83 ± 3.2	81.0 ± 4.2	84.2 ± 3.4	81 ± 1.3
	Sudharshan et al. [61]	2019	None	PLTAS NPMIL	92 ± 5.9	89.1 ± 5.2	87.2 ± 4.3	82 ± 3.0
	Gour et al. [62]	2020	Data augmentation	ResHist Model	82 ± 3.3	88.1 ± 2.7	92.5 ± 2.8	87 ± 2.4
	Lingqiao Li et.al [42]	2018	Data Augmentation, Transfer learning	NDCNN	92.8 ± 2.1	93.9 ± 1.9	93.7 ± 2.2	92.9 ± 1.8
	Gandomkar et.al [63]		Data Augmentation,	ResNET152	94.18 ± 2.1	93.2 ± 1.4	94.7 ± 3.6	93.5 ± 2.9
	Proposed	2021	Stain Normalization	FabNet	99 ± 0.2	89.51 ± 1.7	97.41 ± 1.4	96 ± 1.0

**Table 10:** A comparison of FabNet performance with existing studies on Colorectal histology dataset.

Dataset	Author	Year	Preprocessing	Model		Evaluation Matrices		
Colon (NCT-CRC-HE-100K) dataset					**Accuracy**	**Precision**	**F1 Score**	**Sensitivity**
	Wang et al. [64]	2017	None	BCNN	92.6	91.2	92.8	90.5
	Sari et al. [65]	2018	None	SSAE/SCAE	93.6	93.4	93.2	92.3
	Kather et al. [66]	2019	Stain Normalization	TL+CNN (VGG)	94.3	92.1	93.5	94.1
	Gosh et al. [67]	2021	None	Ensemble DNN	92.8	92.6	92.2	93.1
	Proposed	2021	None	FabNet	98.3	98.3	98.2	98.2

## Data Availability

Publicly available datasets were used in this study. The dataset can be found on www.tubo.tu.ac.kr.

## References

[1] Rahhal M.M.A. (2018). Breast Cancer Classification in Histopathological Images Using Convolutional Neural Network. Int. J. Adv. Comput. Sci. Appl. IJACSA.

[2] Alom M.Z., Yakopcic C., Nasrin M.S., Taha T.M., Asari V.K. (2019). Breast Cancer Classification from Histopathological Images with Inception Recurrent Residual Convolutional Neural Network. J. Digit. Imaging.

[3] Araújo T., Aresta G., Castro E., Rouco J., Aguiar P., Eloy C., Polónia A., Campilho A. (2017). Classification of Breast Cancer Histology Images Using Convolutional Neural Networks. PloS ONE.

[4] Liu Y., Chen C., Wang X., Sun Y., Zhang J., Chen J., Shi Y. (2022). An Epigenetic Role of Mitochondria in Cancer. Cells.

[5] Chen K., Zhang J., Beeraka N.M., Tang C., Babayeva Y.V., Sinelnikov M.Y., Zhang X., Zhang J., Liu J., Reshetov I.V. (2022). Advances in the Prevention and Treatment of Obesity-Driven Effects in Breast Cancers. Front. Oncol..

[6] Chen K., Lu P., Beeraka N.M., Sukocheva O.A., Madhunapantula S.V., Liu J., Sinelnikov M.Y., Nikolenko V.N., Bulygin K.V., Mikhaleva L.M. (2022). Mitochondrial Mutations and Mitoepigenetics: Focus on Regulation of Oxidative Stress-Induced Responses in Breast Cancers. Semin. Cancer Biol..

[7] Xie P., Ma Y., Yu S., An R., He J., Zhang H. (2020). Development of an Immune-Related Prognostic Signature in Breast Cancer. Front. Genet..

[8] Williamson G.R., Plowright H., Kane A., Bunce J., Clarke D., Jamison C. (2020). Collaborative Learning in Practice: A Systematic Review and Narrative Synthesis of the Research Evidence in Nurse Education. Nurse Educ. Pract..

[9] Bardou D., Zhang K., Ahmad S.M. (2018). Classification of Breast Cancer Based on Histology Images Using Convolutional Neural Networks. IEEE Access.

[10] Mccann M.T., Ozolek J.A., Castro C.A., Parvin B., Kovacevic J., Mccann M.T., Member S., Ozolek J.A., Castro C.A., Parvin B. (2014). Automated Histology Analysis: Opportunities for Signal Processing. IEEE Signal Process. Mag..

[11] Robertson S., Azizpour H., Smith K., Hartman J. (2018). Digital Image Analysis in Breast Pathology—From Image Processing Techniques to Artificial Intelligence. Transl. Res..

[12] Ching T., Himmelstein D.S., Beaulieu-Jones B.K., Kalinin A.A., Do B.T., Way G.P., Ferrero E., Agapow P.-M., Zietz M., Hoffman M.M. (2018). Opportunities and Obstacles for Deep Learning in Biology and Medicine. J. R. Soc. Interface.

[13] Iglovikov V., Shvets A. (2018). TernausNet: U-Net with VGG11 Encoder Pre-Trained on ImageNet for Image Segmentation. arXiv.

[14] Raman R., Srinivasan S., Virmani S., Sivaprasad S., Rao C., Rajalakshmi R. (2019). Fundus Photograph-Based Deep Learning Algorithms in Detecting Diabetic Retinopathy. Eye.

[15] Tiulpin A., Thevenot J., Rahtu E., Lehenkari P., Saarakkala S. (2018). Automatic Knee Osteoarthritis Diagnosis from Plain Radiographs: A Deep Learning-Based Approach. Sci. Rep..

[16] Ma X., Niu Y., Gu L., Wang Y., Zhao Y., Bailey J., Lu F. (2021). Understanding Adversarial Attacks on Deep Learning Based Medical Image Analysis Systems. Pattern Recognit..

[17] Sanyal R., Jethanandani M., Sarkar R., Sharma M.K., Dhaka V.S., Perumal T., Dey N., Tavares J.M.R.S. (2021). DAN: Breast Cancer Classification from High-Resolution Histology Images Using Deep Attention Network. Innovations in Computational Intelligence and Computer Vision.

[18] Kumar S., Sharma S. (2022). Sub-Classification of Invasive and Non-Invasive Cancer from Magnification Independent Histopathological Images Using Hybrid Neural Networks. Evol. Intell..

[19] Dou J. (2022). Clinical Decision System Using Machine Learning and Deep Learning: A Survey. https://www.researchgate.net/profile/Jason-Dou/publication/360154101_Clinical_Decision_System_using_Machine_Learning_and_Deep_Learning_a_Survey/links/630b86f5acd814437fe29fe7/Clinical-Decision-System-using-Machine-Learning-and-Deep-Learning-a-Survey.pdf.

[20] Amin M.S., Ahn H. (2021). Earthquake Disaster Avoidance Learning System Using Deep Learning. Cogn. Syst. Res..

[21] Amin M.S., Yasir S.M., Ahn H. (2020). Recognition of Pashto Handwritten Characters Based on Deep Learning. Sensors.

[22] Sadiq A.M., Ahn H., Choi Y.B. (2020). Human Sentiment and Activity Recognition in Disaster Situations Using Social Media Images Based on Deep Learning. Sensors.

[23] Lin M., Chen Q., Yan S. (2014). Network in Network. https://arxiv.org/abs/1312.4400.

[24] Szegedy C., Liu W., Jia Y., Sermanet P., Reed S., Anguelov D., Erhan D., Vanhoucke V., Rabinovich A. (2014). Going Deeper with Convolutions 2014. arXiv.

[25] He K., Zhang X., Ren S., Sun J. (2016). Identity Mappings in Deep Residual Networks. Proceedings of the Computer Vision–ECCV 2016: 14th European Conference.

[26] Srivastava R.K., Greff K., Schmidhuber J. (2015). Highway Networks. arXiv.

[27] Yasrab R. (2019). SRNET: A Shallow Skip Connection Based Convolutional Neural Network Design for Resolving Singularities. J. Comput. Sci. Technol..

[28] Vapnik V.N. (2000). The Nature of Statistical Learning Theory.

[29] Kowal M., Filipczuk P., Obuchowicz A., Korbicz J., Monczak R. (2013). Computer-Aided Diagnosis of Breast Cancer Based on Fine Needle Biopsy Microscopic Images. Comput. Biol. Med..

[30] Spanhol F.A., Oliveira L.S., Petitjean C., Heutte L. (2016). A Dataset for Breast Cancer Histopathological Image Classification. IEEE Trans. Biomed. Eng..

[31] George Y.M., Zayed H.H., Roushdy M.I., Elbagoury B.M. (2014). Remote Computer-Aided Breast Cancer Detection and Diagnosis System Based on Cytological Images. IEEE Syst. J..

[32] Breast Cancer Diagnosis from Biopsy Images with Highly Reliable Random Subspace Classifier Ensembles |SpringerLink. https://link.springer.com/article/10.1007/s00138-012-0459-8.

[33] Robinson E., Silverman B.G., Keinan-Boker L. (2017). Using Israel’s National Cancer Registry Database to Track Progress in the War against Cancer: A Challenge for Health Services. Isr. Med. Assoc. J. IMAJ.

[34] Gupta V., Bhavsar A. Breast Cancer Histopathological Image Classification: Is Magnification Important?. Proceedings of the 2017 IEEE Conference on Computer Vision and Pattern Recognition Workshops (CVPRW).

[35] Seo H., Brand L., Barco L.S., Wang H. (2022). Scaling Multi-Instance Support Vector Machine to Breast Cancer Detection on the BreaKHis Dataset. Bioinformatics.

[36] Rashmi R., Prasad K., Udupa C.B.K. (2021). Breast Histopathological Image Analysis Using Image Processing Techniques for Diagnostic Purposes: A Methodological Review. J. Med. Syst..

[37] Gupta S., Sinha N., Sudha R., Babu C. Breast Cancer Detection Using Image Processing Techniques. Proceedings of the 2019 Innovations in Power and Advanced Computing Technologies (i-PACT).

[38] Das A., Nair M.S., Peter S.D. (2020). Computer-Aided Histopathological Image Analysis Techniques for Automated Nuclear Atypia Scoring of Breast Cancer: A Review. J. Digit. Imaging.

[39] Spanhol F.A., Oliveira L.S., Petitjean C., Heutte L. Breast Cancer Histopathological Image Classification Using Convolutional Neural Networks. Proceedings of the 2016 International Joint Conference on Neural Networks (IJCNN).

[40] Krizhevsky A., Sutskever I., Hinton G.E. (2012). ImageNet Classification with Deep Convolutional Neural Networks. Advances in Neural Information Processing Systems.

[41] Hou L., Samaras D., Kurc T.M., Gao Y., Davis J.E., Saltz J.H. Patch-Based Convolutional Neural Network for Whole Slide Tissue Image Classification. Proceedings of the 2016 IEEE Conference on Computer Vision and Pattern Recognition (CVPR).

[42] Han Z., Wei B., Zheng Y., Yin Y., Li K., Li S. (2017). Breast Cancer Multi-Classification from Histopathological Images with Structured Deep Learning Model. Sci. Rep..

[43] Cireşan D.C., Giusti A., Gambardella L.M., Schmidhuber J., Mori K., Sakuma I., Sato Y., Barillot C., Navab N. (2013). Mitosis Detection in Breast Cancer Histology Images with Deep Neural Networks. Medical Image Computing and Computer-Assisted Intervention—MICCAI 2013.

[44] Wang H., Roa A.C., Basavanhally A.N., Gilmore H.L., Shih N., Feldman M., Tomaszewski J., Gonzalez F., Madabhushi A. (2014). Mitosis Detection in Breast Cancer Pathology Images by Combining Handcrafted and Convolutional Neural Network Features. J. Med. Imaging.

[45] Kashif M.N., Raza S.E.A., Sirinukunwattana K., Arif M., Rajpoot N. Handcrafted Features with Convolutional Neural Networks for Detection of Tumor Cells in Histology Images. Proceedings of the 2016 IEEE 13th International Symposium on Biomedical Imaging (ISBI).

[46] Tellez D., Litjens G., Bándi P., Bulten W., Bokhorst J.-M., Ciompi F., van der Laak J. (2019). Quantifying the Effects of Data Augmentation and Stain Color Normalization in Convolutional Neural Networks for Computational Pathology. Med. Image Anal..

[47] Bejnordi B.E., Zuidhof G., Balkenhol M., Hermsen M., Bult P., van Ginneken B., Karssemeijer N., Litjens G., Laak J. (2017). van der Context-Aware Stacked Convolutional Neural Networks for Classification of Breast Carcinomas in Whole-Slide Histopathology Images. J. Med. Imaging.

[48] Ehteshami Bejnordi B., Mullooly M., Pfeiffer R.M., Fan S., Vacek P.M., Weaver D.L., Herschorn S., Brinton L.A., van Ginneken B., Karssemeijer N. (2018). Using Deep Convolutional Neural Networks to Identify and Classify Tumor-Associated Stroma in Diagnostic Breast Biopsies. Mod. Pathol..

[49] Reinhard E., Ashikhmin M., Gooch B., Shirley P. (2001). Color Transfer between Images. IEEE Comput. Graph. Appl..

[50] Vahadane A., Peng T., Sethi A., Albarqouni S., Wang L., Baust M., Steiger K., Schlitter A.M., Esposito I., Navab N. (2016). Structure-Preserving Color Normalization and Sparse Stain Separation for Histological Images. IEEE Trans. Med. Imaging.

[51] Mathews A., Simi I., Kizhakkethottam J.J. (2016). Efficient Diagnosis of Cancer from Histopathological Images By Eliminating Batch Effects. Procedia Technol..

[52] Kather J.N., Halama N., Marx A. 100,000 Histological Images of Human Colorectal Cancer and Healthy Tissue 2018. https://zenodo.org/record/1214456#.Y98AhK1BxPY.

[53] Macenko M., Niethammer M., Marron J.S., Borland D., Woosley J.T., Guan X., Schmitt C., Thomas N.E. (2009). A Method for Normalizing Histology Slides for Quantitative Analysis. Proceedings of the 2009 IEEE International Symposium on Biomedical Imaging: From Nano to Macro.

[54] Ono Y., Trulls E., Fua P., Yi K.M. (2018). LF-Net: Learning Local Features from Images. Adv. Neural Inf. Process. Syst..

[55] Man R., Yang P., Xu B. (2020). Classification of Breast Cancer Histopathological Images Using Discriminative Patches Screened by Generative Adversarial Networks. IEEE Access.

[56] Simonyan K., Zisserman A. (2014). Very Deep Convolutional Networks for Large-Scale Image Recognition. arXiv.

[57] He K., Zhang X., Ren S., Sun J. Deep Residual Learning for Image Recognition. Proceedings of the IEEE Conference on Computer Vision and Pattern Recognition.

[58] Sheikh T.S., Lee Y., Cho M. (2020). Histopathological Classification of Breast Cancer Images Using a Multi-Scale Input and Multi-Feature Network. Cancers.

[59] Spanhol F.A., Oliveira L.S., Cavalin P.R., Petitjean C., Heutte L. Deep Features for Breast Cancer Histopathological Image Classification. Proceedings of the 2017 IEEE International Conference on Systems, Man, and Cybernetics (SMC).

[60] Kumar K., Rao A.C.S. Breast Cancer Classification of Image Using Convolutional Neural Network. Proceedings of the 2018 4th International Conference on Recent Advances in Information Technology (RAIT).

[61] Sudharshan P.J., Petitjean C., Spanhol F., Oliveira L.E., Heutte L., Honeine P. (2019). Multiple Instance Learning for Histopathological Breast Cancer Image Classification. Expert Syst. Appl..

[62] Gour M., Jain S., Sunil Kumar T. (2020). Residual Learning Based CNN for Breast Cancer Histopathological Image Classification. Int. J. Imaging Syst. Technol..

[63] Gandomkar Z., Brennan P.C., Mello-Thoms C. (2019). Computer-Assisted Nuclear Atypia Scoring of Breast Cancer: A Preliminary Study. J. Digit. Imaging.

[64] Wang C., Shi J., Zhang Q., Ying S. Histopathological Image Classification with Bilinear Convolutional Neural Networks. Proceedings of the 2017 39th Annual International Conference of the IEEE Engineering in Medicine and Biology Society (EMBC).

[65] Sari C.T., Gunduz-Demir C. (2018). Unsupervised Feature Extraction via Deep Learning for Histopathological Classification of Colon Tissue Images. IEEE Trans. Med. Imaging.

[66] Kather J.N., Krisam J., Charoentong P., Luedde T., Herpel E., Weis C.-A., Gaiser T., Marx A., Valous N.A., Ferber D. (2019). Predicting Survival from Colorectal Cancer Histology Slides Using Deep Learning: A Retrospective Multicenter Study. PLoS Med..

[67] Ghosh S., Bandyopadhyay A., Sahay S., Ghosh R., Kundu I., Santosh K.C. (2021). Colorectal Histology Tumor Detection Using Ensemble Deep Neural Network. Eng. Appl. Artif. Intell..

[68] Mewada H.K., Patel A.V., Hassaballah M., Alkinani M.H., Mahant K. (2020). Spectral–Spatial Features Integrated Convolution Neural Network for Breast Cancer Classification. Sensors.

